# Synergistic spatial-sequential modeling for enhanced *Rosa roxburghii* disease detection

**DOI:** 10.1371/journal.pone.0358868

**Published:** 2026-10-06

**Authors:** Xude Zhang, Houbing Tang, Xiaoping Wu, Jianghai Wang

**Affiliations:** 1 Engineering Research Center of Micro-Nano and Intelligent Manufacturing, Ministry of Education, Kaili University, Kaili, Guizhou, China; 2 College of Microelectronics and Artificial Intelligence, Kaili University, Kaili, Guizhou, China; University of Agriculture Faisalabad, PAKISTAN

## Abstract

The rapid advancement of smart agricultural technologies has enabled deep learning–based approaches to markedly enhance the efficiency of crop disease detection and quality monitoring. To overcome the challenges of low detection efficiency and high false-positive rates in large-scale cultivation of the characteristic economic crop *Rosa roxburghii*, this study introduces a synergistic spatial–sequential modeling framework. This synergy is achieved via a gated fusion mechanism that bidirectionally enhances CNN-extracted local spatial features and Mamba-derived global sequential features through adaptive re-weighting, forming a mutual refinement loop instead of a simple cascade. Built upon YOLOv8, the model integrates the Mamba module from state space models (SSMs) to develop a hybrid feature extraction framework, jointly optimizing detection accuracy and inference efficiency. A selective feature enhancement strategy further strengthens the network’s ability to characterize subtle lesions on the surface of *Rosa roxburghii* by adaptively amplifying informative responses through channel-wise recalibration and spatial refinement. The backbone network also incorporates the selective scan for 2D data (SS2D) module to capture long-range dependencies beyond conventional convolution. Experimental validation conducted on our self-constructed dataset, which consists of field-captured images of *Rosa roxburghii* plants manually collected and annotated by the research team, demonstrates that the proposed model delivers notable performance gains, achieving mAP@50 improvements of 6%, 2.87%, 8.38%, 7.08%, 16.27%, and 6.55% over YOLOv5-N, YOLOv8-N, YOLOv10-N, YOLOv11-N, YOLOv12-N, and MambaYOLO-T, respectively. Concerning mean average precision (mAP)@ 50–95, the model attains corresponding gains of 6.85%, 0.27%, 6.99%, 5.19%, 13.66%, and 2.57%, all while maintaining comparable FLOPs (G) and parameter counts. These results highlight the model’s excellent performance, confirming its effectiveness for agricultural visual inspection and suggesting a promising approach for intelligent disease monitoring in mountainous specialty crop production.

## Introduction

With the continuous progression of agricultural modernization and the rapid evolution of artificial intelligence, the agricultural industry is experiencing a profound transformation [1]. Guizhou, recognized as a primary production region for *Rosa roxburghii*, relies heavily on the *Rosa roxburghii* industry as a driving force for its regional economic growth [2]. However, recurrent disease outbreaks severely affect both the quantity and quality of agricultural output [3]. Traditional approaches to *Rosa roxburghii* disease detection often rely on manual experience and handcrafted features, which fall short when confronted with highly variable disease manifestations and diverse field conditions, leading to limited detection accuracy and efficiency [4]. The rapid development of deep learning technologies has introduced promising avenues for *Rosa roxburghii* image disease detection [5]. Specifically, integrating a Mamba mechanism into a feature learning-based model enables automatic extraction of high-level disease-related patterns from *Rosa roxburghii* images, addressing the limitations of manually designed features [6]. This enhanced model demonstrates strong robustness and generalization capabilities under varying lighting conditions, angles, and disease development stages [7], with real-time processing capability meeting the practical demands of agricultural field response. Therefore, this study investigates a Mamba-enhanced feature learning approach for *Rosa roxburghii* disease detection, aiming to provide scientific and technical support for intelligent disease management in the *Rosa roxburghii* industry.

With the growing integration of artificial intelligence into agriculture, deep learning-based methods for disease detection technology in *Rosa roxburghii* have become a critical area of research direction for promoting sustainable development [8]. Traditional image processing techniques, which rely on manually crafted features. They struggle to handle complex leaf lesion morphology, color gradients, and background interference in real-world field environments. Recent advances in UAV and hyperspectral-based crop monitoring have demonstrated the value of multi-layer sensing approaches for assessing plant growth and stress responses [9]. In contrast, deep learning significantly improves disease identification by automatically learning multi-level feature representations [10]. Convolutional neural networks (CNNs) extract local texture features through stacked convolutional and pooling layers, yet their fixed receptive fields limit the modeling of cross-regional disease diffusion patterns [11]. Transformer-based vision models address this issue by employing self-attention mechanisms to capture global contextual information; however, they demand substantial data and computational resources, hindering their use on edge devices [12]. Hybrid architecture models that integrate CNNs and Transformers overcome these limitations by effectively fusing local and global feature extraction to enhance detection accuracy. Nevertheless, existing methods continue to face challenges with information redundancy in modeling long-range dependencies [13]. To address the minute, sparse, and morphologically diverse lesions in *Rosa roxburghii* disease images, this study introduces a Hybrid Mamba convolutional vision network. AI-driven frameworks have been increasingly adopted for plant stress management through computer vision and data analytics [14]. The core novelty is a synergistic spatial-sequential fusion mechanism where the CNN components extract local spatial features and the VSSBlock with SS2D captures global sequential features. These streams are fused via a gated mechanism where sequential features generate importance weights to adaptively enhance spatial features through element-wise multiplication, creating a bidirectional refinement loop for mutual enhancement.Integrating the Mamba state space model (SSM) with CNNs, it selectively updates states to link local lesion features with cross-regional diffusion patterns while maintaining CNN efficiency. This approach enables real-time monitoring and precise control of *Rosa roxburghii* diseases [15–17].

This study presents YOLO-HMC, a Hybrid Mamba-convolutional model for detecting diseases in *Rosa roxburghii* images. We select YOLOv8 as the baseline architecture for three key reasons: its established balance between detection accuracy and inference speed, its modular design that readily accommodates the integration of the Mamba module, and its extensive validation across agricultural detection tasks which provides a reliable reference for performance comparison. While newer YOLO versions offer incremental improvements, their architectural changes often introduce additional complexity that may obscure the evaluation of our proposed VSSBlock enhancement. By embedding the Mamba mechanism into the YOLOv8 detection framework, we build a hierarchical feature extractor that enables precise, real-time detection of lesion areas on *Rosa roxburghii* leaves and fruit. While YOLO-based detectors are valued for their end-to-end detection efficiency, they face major challenges with complex *Rosa roxburghii* diseases. Traditional convolution operations struggle to capture fine spatial features of minute lesions, causing frequent missed detection rates, while single attention mechanisms fail to model lesion spread across regions. Existing methods also lack robustness in distinguishing multi-scale overlapping lesions. To overcome these limitations, we develop the Mamba-based VSS module that integrates state-space transformations into feature extraction. By dynamically constructing pixel-level state transition matrices, the module captures the radial diffusion of anthracnose lesions and the fractal growth of powdery mildew. The overall model forms a “local-global” perception system in which the bottom layer employs an improved CNN for rapid extraction of edge and texture features, the middle layer captures cross-regional spatial correlations through Mamba, and the top layer integrates a hybrid CNN with the Mamba approach to achieve semantic fusion.

- First construction of a *Rosa roxburghii* disease image dataset containing 5,063 annotated images across seven disease categories.
- Introduction of a CNN-Mamba module-enhanced architecture to overcome spatial modeling limitations in YOLO-based agricultural disease detection.
- Demonstration of the effective integration of the Mamba mechanism within the YOLO framework, offering a novel approach for intelligent monitoring of specialty economic crops.

## Related work

### Traditional method-based image detection of *Rosa roxburghii*

As a high-value specialty crop, *Rosa roxburghii* Tratt requires accurate detection of surface lesions to ensure optimal yield and quality. Traditional image processing methods primarily rely on extracting and classifying lesion features in *Rosa roxburghii* fruit images, employing detection methods based on color space analysis, texture feature extraction, and morphological operations. In particular, color space analysis reveals distinct differences in color distribution between lesion regions and healthy tissues on the *Rosa roxburghii* fruit surfaces [18]. To mitigate the effects of uneven natural environments, research employs the Top-Hat Transform for shadow removal with median filtering to suppress noise interference [19]. Images are converted to the HSV color space, and threshold segmentation is applied to the H channel to isolate lesion regions under consistent lighting. For overlapping fruits, the watershed algorithm combined with the color difference analysis enables accurate detection and localization, even under occlusion or crowding conditions, enhancing segmentation accuracy [20]. Additionally, texture feature extraction plays a crucial role in differentiating lesions from healthy tissue. The Gray-Level Co-occurrence Matrix quantifies image texture by calculating the gray-level relationships of pixel pairs across various orientations and distances, capturing features, such as roughness and contrast within lesion regions. To mitigate textural blurring caused by mildew on *Rosa roxburghii* fruit surfaces, studies extract GLCM feature parameters, including energy and entropy, combined with a support vector machine for classification, yielding high accuracy on test datasets [21]. Within the test set containing images, this method achieves favorable classification accuracy. Furthermore, Local binary patterns and their variants enhance the detection sensitivity to subtle lesions by comparing the gray-level relationships between central pixels and their neighbors [22].

### Deep learning-based image detection of *Rosa roxburghii*

Recent advancements in deep learning have significantly improved *Rosa roxburghii* disease detection, addressing the limitations of traditional image processing techniques in complex field environments. CNNs are widely applied in *Rosa roxburghii* disease identification due to their powerful local feature extraction capabilities. Enhanced models, such as an improved YOLOv3 integrated with residual modules, have shown promising results in real-time and accurate *Rosa roxburghii* fruit recognition in natural environments [23]. However, traditional CNNs are limited by fixed receptive fields, making them less effective in capturing the long-range and cross-regional spread of *Rosa roxburghii* diseases [24]. To overcome this issue, the Transformer architectures have been introduced, leveraging self-attention mechanisms to model global lesion correlations. Nevertheless, its high computational complexity and reliance on large-scale data training pose challenges for deployment on edge devices [25]. Recently, SSMs, especially the Mamba mechanism, have emerged as efficient alternatives for *Rosa roxburghii* disease detection, offering linear computational complexity and selective state updates. The VMUnet-MSADI model, which integrates visual state space modules, multi-scale attention mechanisms, and detail injection blocks [26], demonstrates superior accuracy in agricultural image segmentation tasks compared to traditional U-Net [27]. InsectMamba integrates SSMs, CNNs, and self-attention mechanisms to effectively fuse multimodal features through hybrid SSM blocks, achieving strong results in pest classification and demonstrating the broad applicability of SSMs in agricultural vision [28]. By leveraging CNNs for local feature extraction and Mamba for long-range dependencies, this approach enhances the accuracy and efficiency of *Rosa roxburghii* disease detection, providing reliable technical support for smart agriculture [29,30]. However, these prior methods do not fully exploit bidirectional feature enhancement. In contrast, our YOLO-HMC introduces three key innovations beyond the VSSBlock: a synergistic spatial-sequential fusion mechanism that bidirectionally enhances local and global features, a selective feature enhancement strategy that adaptively amplifies lesion responses, and a gated fusion mechanism for iterative feature refinement. These components collectively address the multi-scale and complex background challenges inherent in agricultural disease detection.

## Method

### Preliminaries

This section introduces the foundational concepts of SSMs [31], which form the foundation of YOLO-HMC. SSMs, such as S4 and Mamba Dare, are based on discretizing continuous systems to model sequential data. These models construct an implicit latent state space to progressively map input sequences x(t)∈R to output sequences y(t). The system first maps discrete input sequences into a continuous latent space $h^{\prime }\left(t\right)\in R^{N}$ using a parameterized state transition matrix A, and models dynamics through the state and observation equations. The S4 model enables efficient training via fast convolution kernel computation, while Mamba enhances expressiveness by introducing input-dependent dynamic parameters. This discretized continuous-system approach effectively captures long-range dependencies and improves computational efficiency through structured parameterization. The mathematical definition of the system can be summarized as follows:


$$\begin{array}{c} h^{\prime }\left(t\right)=Ah\left(t\right)+Bx\left(t\right) \end{array}$$ (1)

$$\begin{array}{c} y\left(t\right)=Ch\left(t\right) \end{array}$$ (2)

The Mamba model introduces a novel time scale parameter 𝛥 to discretize continuous state-space models for improved integration with deep learning frameworks. It uses a zero-order hold technique to transform the continuous parameters A and B into their discrete counterparts, $\overset{―}{A}$ and $\overset{―}{B}$, following a precise discretization rule:


$$\begin{array}{c} \overset{―}{A}=\exp\left(\Delta A\right) \end{array}$$ (3)

$$\begin{array}{c} \overset{―}{B}={\left(\Delta A\right)}^{-1}\left(\exp\left(\Delta A\right)-I\right)\Delta B \end{array}$$ (4)

where $\overset{―}{A}$ and $\overset{―}{B}$ denote the discrete-time equivalents of the continuous parameters A and B, respectively, capturing their dynamics across the predetermined sampling period 𝛥. The identity matrix I ensures dimensional consistency throughout the conversion process.

After discretization, the model operates via a linear recurrence given by:


$$\begin{array}{c} h^{\prime }\left(t\right)=\overset{―}{A}h_{t-1}+\overset{―}{B}x_{t} \end{array}$$ (5)

Alternatively, the model’s sequential computation can be expressed as a global convolution of the input sequence with a structured kernel determined by the SSM parameters. This convolutional representation is mathematically expressed as:


$$\begin{array}{c} y=x*\overset{―}{K} \end{array}$$ (6)

where $\overset{―}{K}\in R^{L}$ indicates the SSM convolution kernel, and *L* signifies the length of the input sequence *x*.


$$\begin{array}{c} \overset{―}{K}=\left(C\overset{―}{B},C\overset{―}{AB},....,C{\overset{―}{A}}^{L-1}\overset{―}{B}\right) \end{array}$$ (7)

### Overall architecture

This section presents the architecture of YOLO-HMC, as illustrated in Fig 1. The model comprises a backbone, PAFPN, and a detection head. The Backbone, built from Conv Blocks and VSS Blocks, extracts features, with each Conv Block containing convolutional layers, batch normalization, and activation function layers. The PAFPN primarily consists of C2f Blocks, concatenation, and upsampling layers, enabling multi-scale feature fusion to enhance detail capture and contextual relationships, which boosts detection accuracy. Finally, the detection head predicts the bounding boxes, categories, and confidence scores for targets within the image.

**Fig 1:**
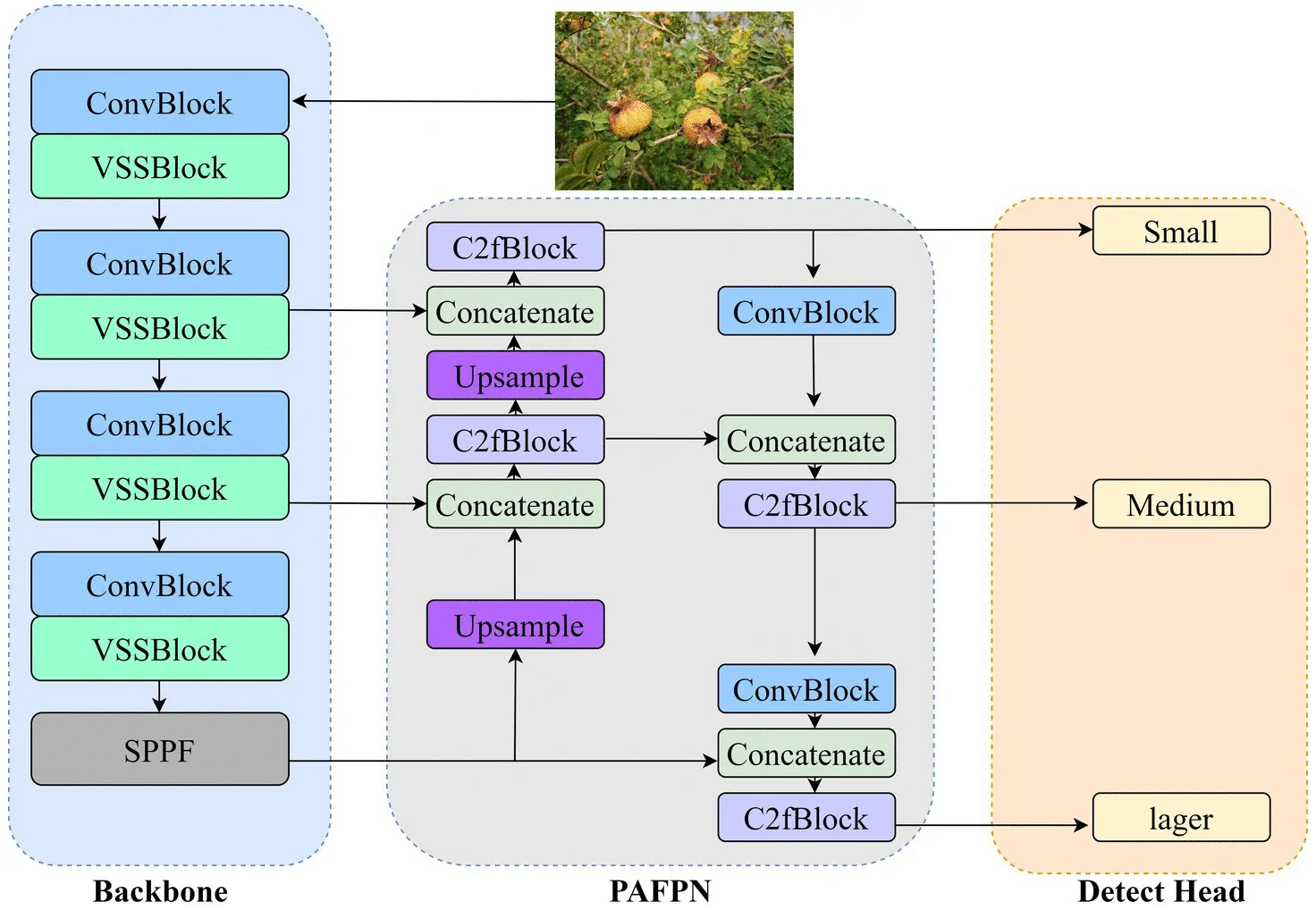
Overview of the YOLO-HMC architecture.

### C2f Block

Fig 2 illustrates the C2f Block, which consists of an input convolutional layer, feature splitting, multiple Bottleneck blocks, and a final convolutional layer. Its key innovation employs a cross-stage partial fusion strategy: the feature maps are split along the channel dimension into two pathways. One pathway processes deep semantic features through stacked bottleneck blocks, while the other retains shallow spatial details. These multi-scale features are fused by concatenation. This design shortens gradient propagation paths and boosts feature reuse rates, reducing parameters and computation without sacrificing accuracy. As a result, the module enhances inference speed, resource utilization, and overall detection accuracy and robustness.

**Fig 2:**
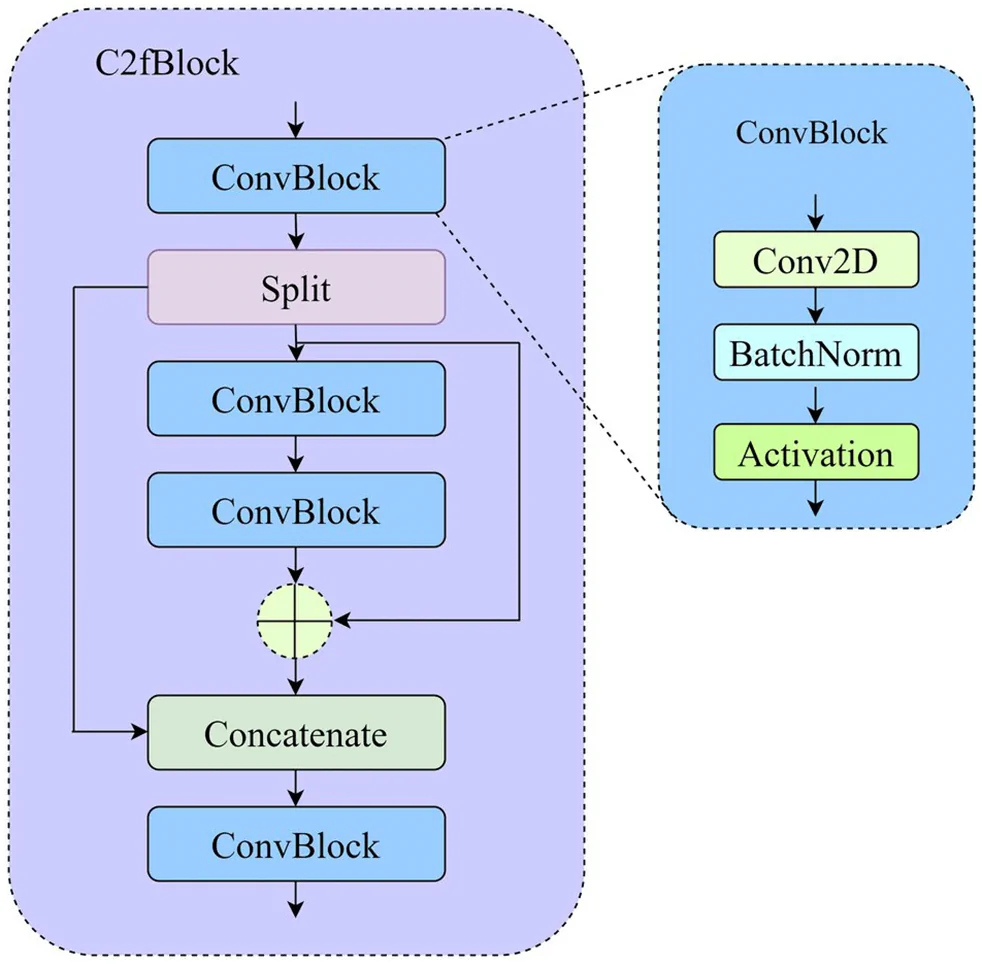
Overview of the C2f block architecture.

The C2f block significantly reduces computational complexity by selectively processing features to reduce redundancy and boost feature diversity. By effectively combining shallow features and deep abstract features, it enhances feature representation capability. Additionally, it improves gradient flow, helping to prevent vanishing gradients in deep networks.

### Synergistic spatial-sequential fusion mechanism

The synergy is defined as bidirectional enhancement between spatial and sequential features rather than a simple cascade. The YOLOv8 backbone and C2f blocks extract spatial features $F_{CNN}$,while the VSSBlock with SS2D captures sequential features $T^{\prime }$.The synergy occurs via gated fusion at the C2f-VSSBlock interface:


$$\begin{array}{c} W=\sigma (\text{Linear}(T^{\prime })),\;F_{fused}=W⊙F_{CNN} \end{array}$$ (8)

where σ is sigmoid, ⊙ is element-wise multiplication, and W are importance weights.This fusion executes after each VSSBlock stage and creates a bidirectional loop where $F_{fused}$ feeds to subsequent VSSBlock stages, iteratively refining both representations. This mutual enhancement constitutes the synergy in our title.

### VSSBlock

Fig 3 highlights the VSSBlock as the core component of the YOLO-HMC model, playing a critical role in enhancing the model’s overall feature extraction capabilities while maintaining high processing efficiency and throughput. It introduces the scanning selection in the two dimensions (SS2D) module, which uses a novel four-directional scanning strategy to overcome the limitations in traditional feature extraction methods [32]. As shown in Fig 4, the SS2D module scans the feature map concurrently from the top-left, top-right, bottom-left, and bottom-right corners. This parallelized scanning allows each feature element to gather information from all other positions via multiple paths, effectively capturing multi-scale features. During scanning, the SS2D module preserves the image’s original spatial structure and enhances its ability to capture image details through independent directional scans. Scanning paths from multiple orientations capture features across varying scales, ranging from fine local details to broad global structures, thereby forming a hierarchical feature representation. The SS2D module then cleverly merges one-dimensional vectors obtained from these directions into a compact two-dimensional feature output using a specialized fusion method. This process reduces feature redundancy, improves the compactness of feature representation, and boosts feature quality, leading to more precise target identification in later stages.

**Fig 3:**
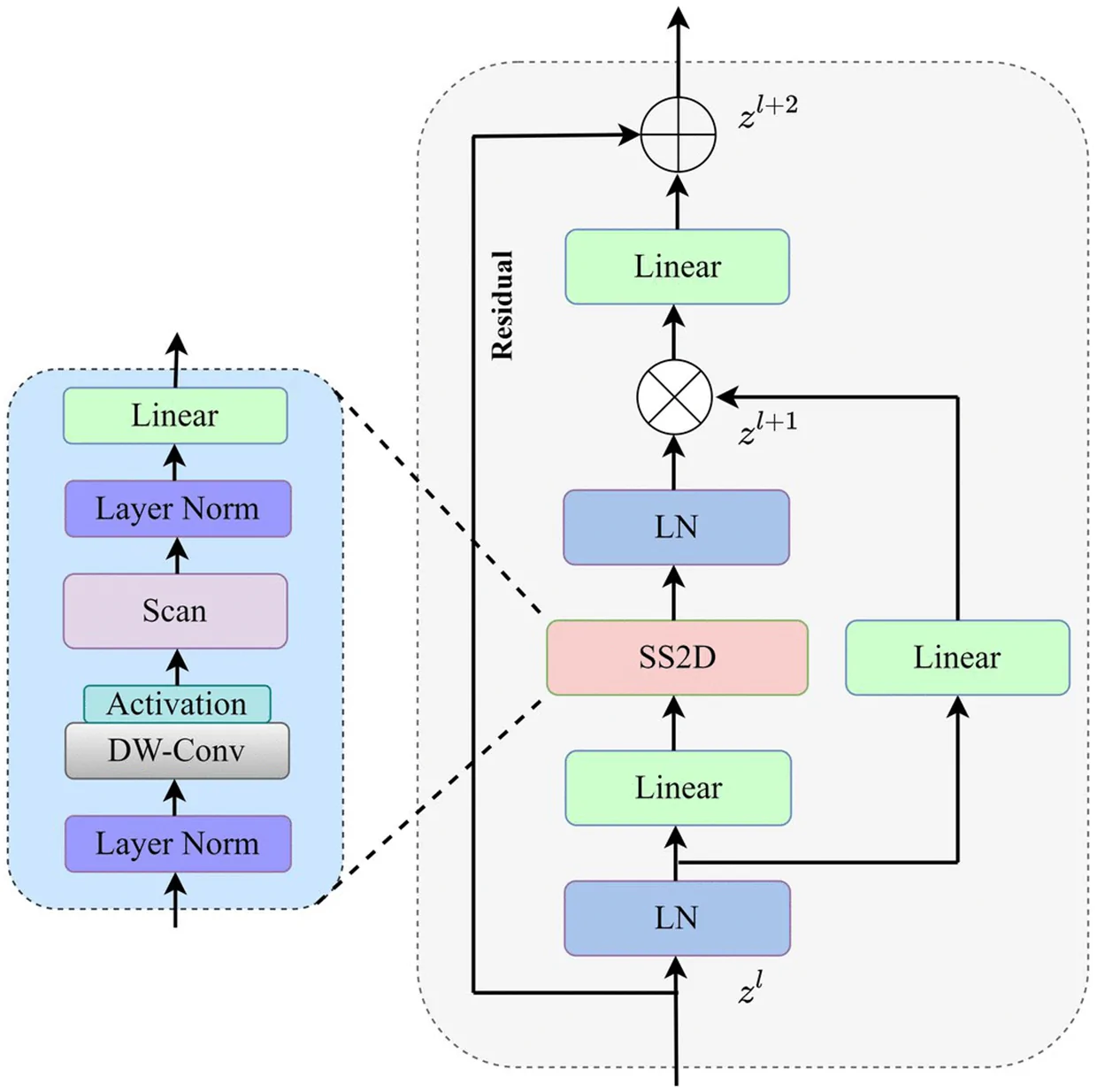
Overview of the VSS block.

**Fig 4:**
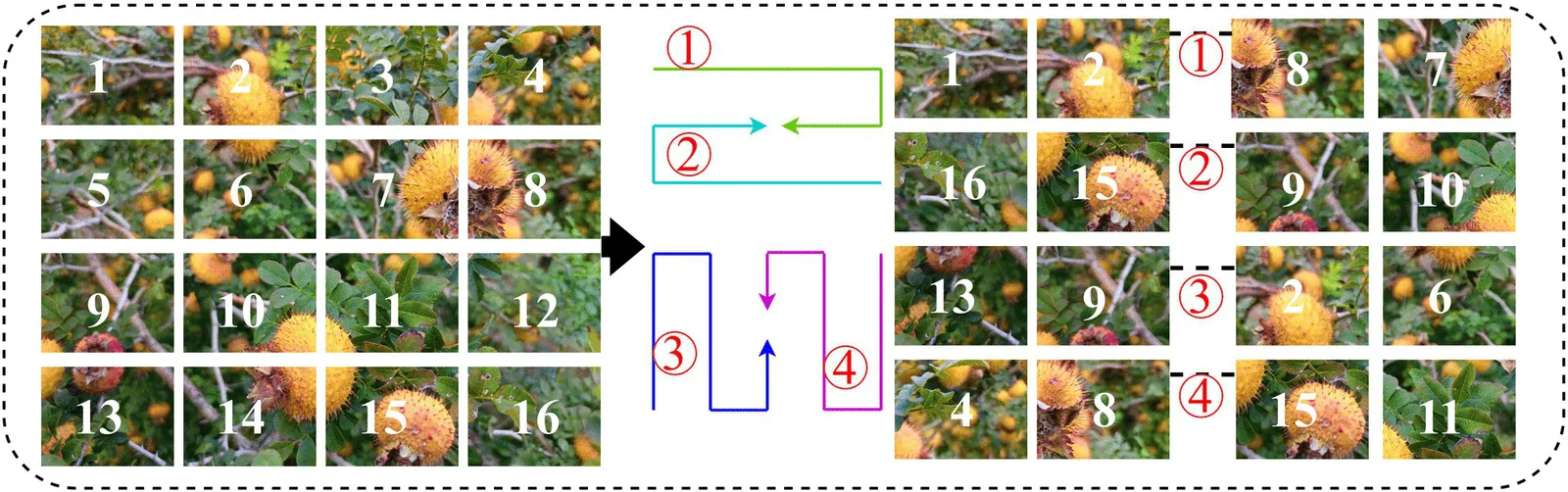
Scanning process of the SS2D module.

The VSSBlock architecture is inspired by Transformer blocks, featuring layer normalization and residual connections. This design ensures stable operation even with deep stacking, preventing performance degradation during training in deeply stacked network architectures.

where $Z^{l}$ and $Z^{l+2}$ denote the input and output features, respectively, and $Z^{l+1}$ represents the intermediate state after 2D-Selective-Scan. Their computational formulas are defined as follows:


$$\begin{array}{c} Z^{l+1}=LN\left(SS2D\left(Lin\left(LN\left(z^{l}\right)\right)\right)\right)\times Lin\left(LN\left(Z^{l}\right)\right) \end{array}$$ (9)

$$\begin{array}{c} Z^{l+2}=Z^{l}+Lin\left(Z^{l+1}\right) \end{array}$$ (10)

The SS2D module in VSSBlock offers key benefits: it employs a four-directional parallel scanning approach to efficiently capture multi-scale features while maintaining the spatial structure of the image. Independent scans from four directions improve detail extraction and create hierarchical feature representations. A smart merging step consolidates multi-directional one-dimensional vectors into compact two-dimensional features, minimizing redundancy and boosting feature quality. This configuration enables more precise target identification and substantially improves the overall model performance.

### Selective feature enhancement strategy

To strengthen the network’s ability to characterize subtle lesions, we introduce a selective feature enhancement strategy that adaptively amplifies informative responses while suppressing irrelevant background activations. This strategy operates on the fused feature maps $F_{fused}$ produced by the synergistic fusion mechanism. The enhancement process consists of three steps: First, a global attention vector is computed by applying global average pooling to $F_{fused}$, followed by two fully connected layers with a reduction ratio r to capture channel-wise dependencies:


$$\begin{array}{c} g=\sigma (W_{2}\delta (W_{1}\text{GAP}(F_{fused}))) \end{array}$$ (11)

where GAP denotes global average pooling,*W*_1_ and *W*_2_ are learnable weights,δ is ReLU, and σ is sigmoid. Second, the attention vector g is used to recalibrate the feature map through channel-wise multiplication:


$$\begin{array}{c} F_{enhanced}=g⊙F_{fused} \end{array}$$ (12)

Third, a spatial refinement module further emphasizes lesion-relevant regions by applying a lightweight convolutional layer with kernel size 3x3 to generate spatial attention weights:


$$\begin{array}{c} A_{spatial}=\text{Conv}3\times 3(Fenhanced),\;F_{final}=A_{spatial}⊙F_{enhanced} \end{array}$$ (13)

This selective enhancement strategy enables the network to focus on subtle lesion features such as mildew spots and scabs while suppressing irrelevant background noise, thereby improving detection accuracy for fine-grained disease patterns.

## Experiments

This section presents a systematic experimental evaluation of the Mamba-enhanced approach for the *Rosa roxburghii* image disease detection. Specifically, we evaluated the detection performance of this approach on both *Rosa roxburghii* leaf disease and fruit defect datasets to verify its robustness in complex agricultural scenarios.

### Environment and hyperparameters

The experimental setup is detailed in Table 1. To address these common challenges, such as uneven illumination and complex backgrounds in agricultural images, data augmentation is employed to improve model generalization. The model is trained from scratch to avoid biases from pre-trained models on natural images, enabling it to better learn fine-grained features of *Rosa roxburghii* lesions. Training is conducted for 100 epochs using a stochastic gradient descent (SGD) optimizer, with an initial learning rate of 0.01 and batch size of 4, both adjusted dynamically based on validation performance. Early stopping is introduced to prevent overfitting, saving model weights only when validation accuracy improves,All experiments are repeated five times with different random seeds, and the results are reported as mean ± standard deviation to ensure statistical reliability. For the N and M variants, the standard deviations for mAP50-95 are consistently below 0.06% and 0.05% respectively, confirming that the reported improvements are statistically consistent across multiple runs.

**Table 1:** Configuration of the experimental environment.

Category	Conffguration
CPU	16 vCPU Intel(R) Xeon(R) Platinum 8474C
GPU	RTX 4090D(24GB)
System environment	ubuntu20.04
Framework	PyTorch 2.0.0
Programming Languages	Python 3.8

### Datasets

This study uses the *Rosa roxburghii* object dataset (RrOD), which includes 5,063 carefully annotated and validated, representing diverse natural environments of *Rosa roxburghii* cultivation. As shown in Fig 5, the dataset captures a wide range of natural environmental scenarios for *Rosa roxburghii* growth, including open-air plantation, field, rainy weather, and intense illumination conditions. The images were acquired from multiple cultivation sites across Guizhou province, covering three major production regions with varied topographical and climatic conditions. Each image was professionally annotated by trained plant pathologists using a polygonal labeling tool to precisely delineate lesion boundaries. All annotations were independently reviewed and verified by experts to ensure labeling consistency and accuracy. The dataset covers seven object categories: leaf lesion, fruit pest infestation, fruit disorder, mature fruit, foliar pest infestation, insect, and immature fruit. The class distribution shows a balanced representation across all categories, ensuring sufficient data for effective model training across all disease types.To capture the complexity of *Rosa roxburghii* cultivation environments, the RrOD dataset features images taken under various environmental conditions, such as sunny, cloudy, and rainy weather, as well as images captured during different times of day and lighting levels, reflecting real-world field variability. The dataset covers diverse growth stages of *Rosa roxburghii* plants and includes variations in disease severity, providing comprehensive coverage for robust model evaluation.The dataset features a modular design, allowing researchers to add new lesion categories or incorporate images from various production regions as needed, supporting future research expansion. All images were professionally annotated and thoroughly validated to ensure data reliability.

**Fig 5:**
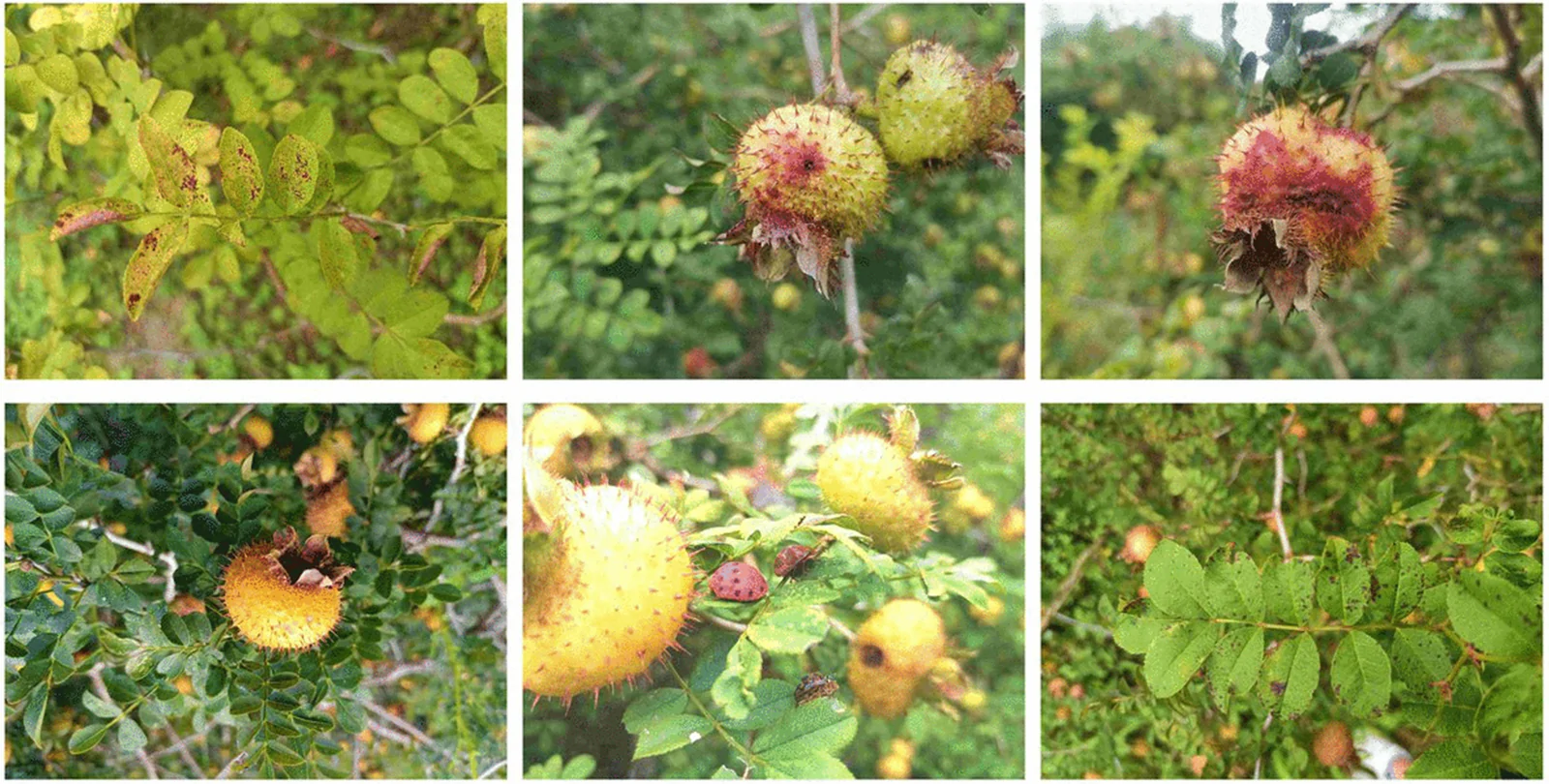
Sample images from the *Rosa roxburghii* object dataset.

### Models training

To systematically validate the effectiveness of the proposed Mamba mechanism-based *Rosa roxburghii* image disease detection method, YOLOv8, widely adopted in agriculture, is adopted as the baseline model. Four scale-matched variants (N/S/M/L) are constructed, and all models are trained for 100 epochs with a batch size of 4 under identical hyperparameters.

The experiments follow a controlled-variable approach, comparing the proposed YOLO-HMC model against mainstream detection models including YOLOv5 [33], YOLOv8 [34], YOLOv10 [35], YOLOv11 [36], YOLOv12 [37], and Mamba YOLO [38] under uniform evaluation standards to ensure fair comparison. For *Rosa roxburghii* image detection tasks, the evaluation metric system must simultaneously capture both sensitivity to subtle lesions and resilience to background noise. This study evaluates model performance and deployment efficiency using Parameters (M) and FLOPs (G), where lower values indicate superior deployment suitability, and mean average precision (mAP), where higher values signify better detection performance.

The progression of mAP@0.5 and mAP@0.5:0.95 for YOLO-HMC during 100 epochs of training is shown in Fig 6, while training loss evolution is depicted in Fig 7.

**Fig 6:**
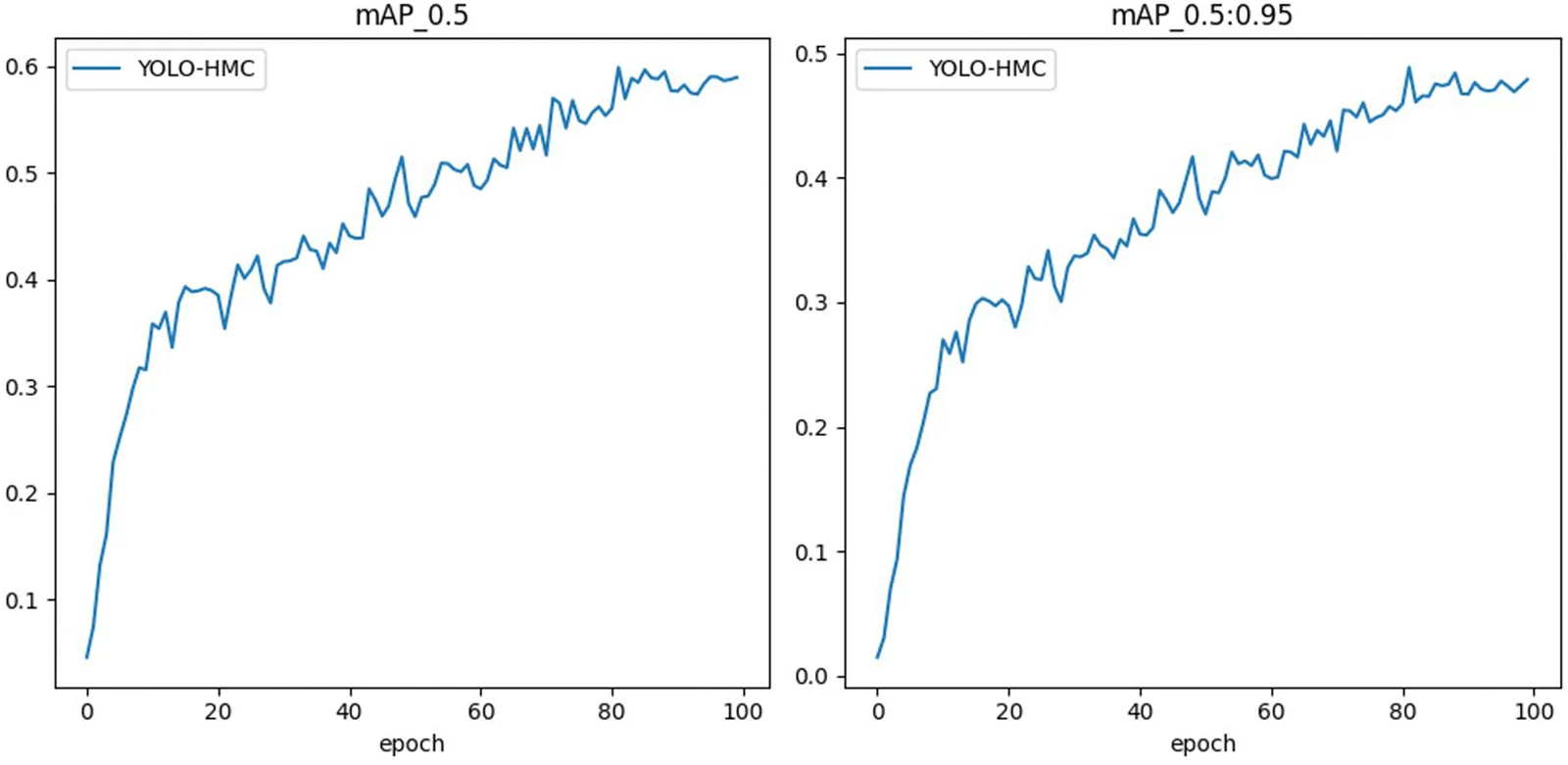
Variation of mAP in the YOLO-HMC model.

**Fig 7:**
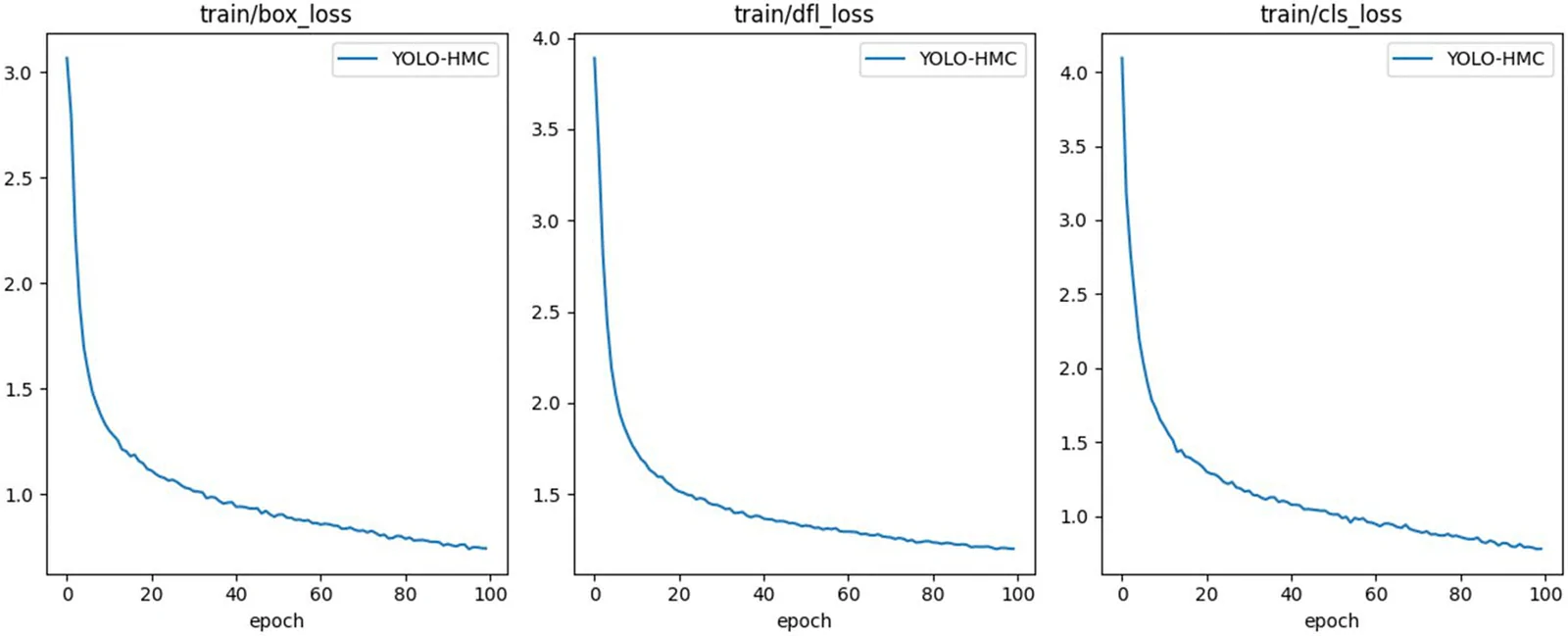
Training loss variation of the YOLO-HMC model.

As illustrated in Fig 8, the normalized confusion matrix highlights the strong performance of our proposed synergistic spatial-sequential modeling in *Rosa roxburghii* disease detection. It achieves over 75% accuracy in key categories, such as leaf lesions, fruit disorders, and mature fruits, reflecting effective feature extraction of major pathological traits. This setup demonstrates the network’s strong capability to extract key pathological features. After normalization, inter-class relationships become more distinct, with color gradients visually highlighting classification limitations in borderline cases.

**Fig 8:**
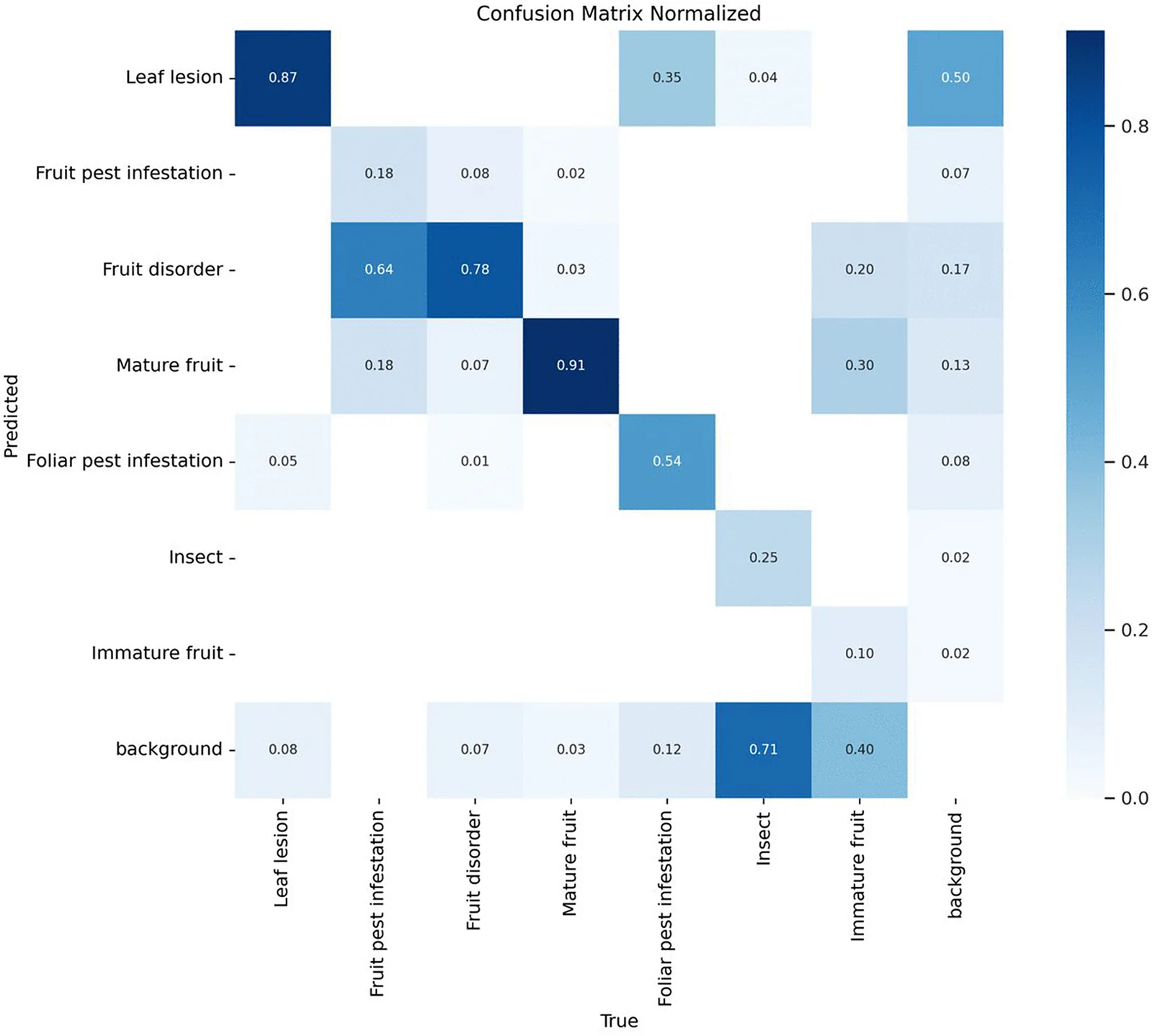
Overview of the confusion matrix.

The per-class precision, recall, and F1-score derived from the confusion matrix in Fig 8 have been thoroughly analyzed. For the pest and disease categories, the model demonstrates strong performance with precision/recall/F1 of 0.87/0.49/0.63 for leaf lesion, 0.78/0.43/0.55 for fruit disorder, 0.54/0.79/0.64 for foliar pest infestation, and 0.25/0.93/0.39 for insect. Notably, the model achieves excellent recall of 0.93 for insect and 0.79 for foliar pest infestation, confirming its reliable detection capability for these visually challenging pest categories. The high precision of 0.87 for leaf lesion and 0.78 for fruit disorder further demonstrates the model’s accuracy in identifying key disease types.

### Ablation study

This section presents an ablation study of the VSSBlock modules within YOLO-HMC on the RrOD dataset. Each component is independently evaluated to assess its impact on Rr image detection. Table 2 shows performance variations with different module configurations (“YES” for active, “NO” for inactive). Metrics include Parameters (M) and FLOPs (G), where lower values indicate better deployment efficiency, and mean Average Precision (mAP), where higher values reflect improved detection accuracy.

**Table 2:** Ablation study on the YOLO-HMC model.

Model	Linear	LN	SS2D	mAP50(%)	mAP75(%)	mAP50-95(%)
1	YES	YES	NO	56.57	53.35	47.28
2	YES	NO	YES	56.5	53.3	48.3
3	NO	YES	YES	57.6	54.13	48.4
4(Ours)	YES	YES	YES	59.9	54.28	48.68

### Quantitative comparisons with state-of-the-art *Rosa roxburghii* disease detection methods

Table 3 presents comparative results of our YOLO-HMC model against other image detection models on the RrOD dataset, evaluating the proposed method through metrics including mAP50-95(%), mAP50(%), mAP75(%), FLOPs(G), and Param.(M). YOLO-HMC outperforms nearly all reference models on the RrOD dataset, Fig 9 illustrates the detection results of disease on *Rosa roxburghii* using our model, while Fig 10 provides a comparative visualization of detection outcomes obtained by other models. From the detection effects shown in the two figures, it can be observed that our model successfully identifies Mature fruit, Leaf lesion, and Fruit disorder. In contrast, among the other models, only YOLOv8, YOLOv11, and YOLOv12 are capable of detecting Fruit disorder. However, none of the other models accurately recognize the Leaf lesion feature. This superior performance stems from the synergistic spatial-sequential fusion mechanism, which enables the model to capture both fine-grained texture details and global context. The selective feature enhancement further improves the detection of subtle lesions frequently missed by other YOLO variants. Therefore, our model not only demonstrates high detection accuracy but also excels in correctly detecting fine-grained features.

**Table 3:** Comparison of YOLO-HMC with other *Rosa roxburghii* disease detection approaches on the RrOD datasets. (Bold indicates the proposed framework).

Framework	#Param.	FLOPs	mAP50-95	mAP50	mAP75
	(M)	(G)	(%)	(%)	(%)
YOLOv5-N [33]	2.5	7.1	45.56	56.51	50.47
YOLOv8-N [34]	3.1	8.1	48.55	58.23	54.48
YOLOv10-N [35]	2.3	6.5	45.5	55.27	50.87
YOLOv11-N [36]	2.6	6.3	46.28	55.94	52.01
YOLOv12-N [37]	2.5	5.8	42.83	51.52	48.27
Mamba YOLO-T [38]	5.9	13.6	47.46	56.22	53
**YOLO-HMC-N (Ours)**	**3.1**	**7.9**	**48.68**	**59.9**	**54.28**
YOLOv5-S [33]	9.1	23.8	48.81	59.27	55.71
YOLOv8-S [34]	11.2	28.5	49.42	59.59	55.03
YOLOv10-S [35]	7.2	21.4	44.55	53.51	50.7
YOLOv11-S [36]	9.4	21.3	49.27	60.1	55.03
YOLOv12-S [37]	9.1	19.3	50.01	59.79	56.35
**YOLO-HMC-S (Ours)**	**11.2**	**27.6**	**52.19**	**63.88**	**56.41**
YOLOv5-M [33]	25.1	64	54.04	64.21	59.44
YOLOv8-M [34]	25.9	78.7	54.44	66.03	59.27
YOLOv10-M [35]	15.3	58.9	46.04	55.61	51.24
YOLOv11-M [36]	20	67.7	52.03	62.31	57.01
YOLOv12-M [37]	19.6	59.5	49.67	59.04	55.34
Mamba YOLO-B [38]	21.8	49.6	52.85	63.06	58.38
**YOLO-HMC-M (Ours)**	**25.9**	**75.2**	**54.54**	**65.06**	**59.79**
YOLOv5-L [33]	53.1	134.7	52.82	62.91	57.42
YOLOv8-L [34]	43.6	164.8	54.3	65.73	59.07
YOLOv10-L [35]	24.3	120	44.3	54.3	49.64
YOLOv11-L [36]	25.2	86.6	53.55	64.57	57.79
YOLOv12-L [37]	25.8	80.8	50.6	61.17	56.55
Mamba YOLO-L [38]	57.6	155.9	53.65	64.1	60.57
**YOLO-HMC-L (Ours)**	**43.6**	**155.7**	**53.32**	**63.59**	**61.01**

**Fig 9:**
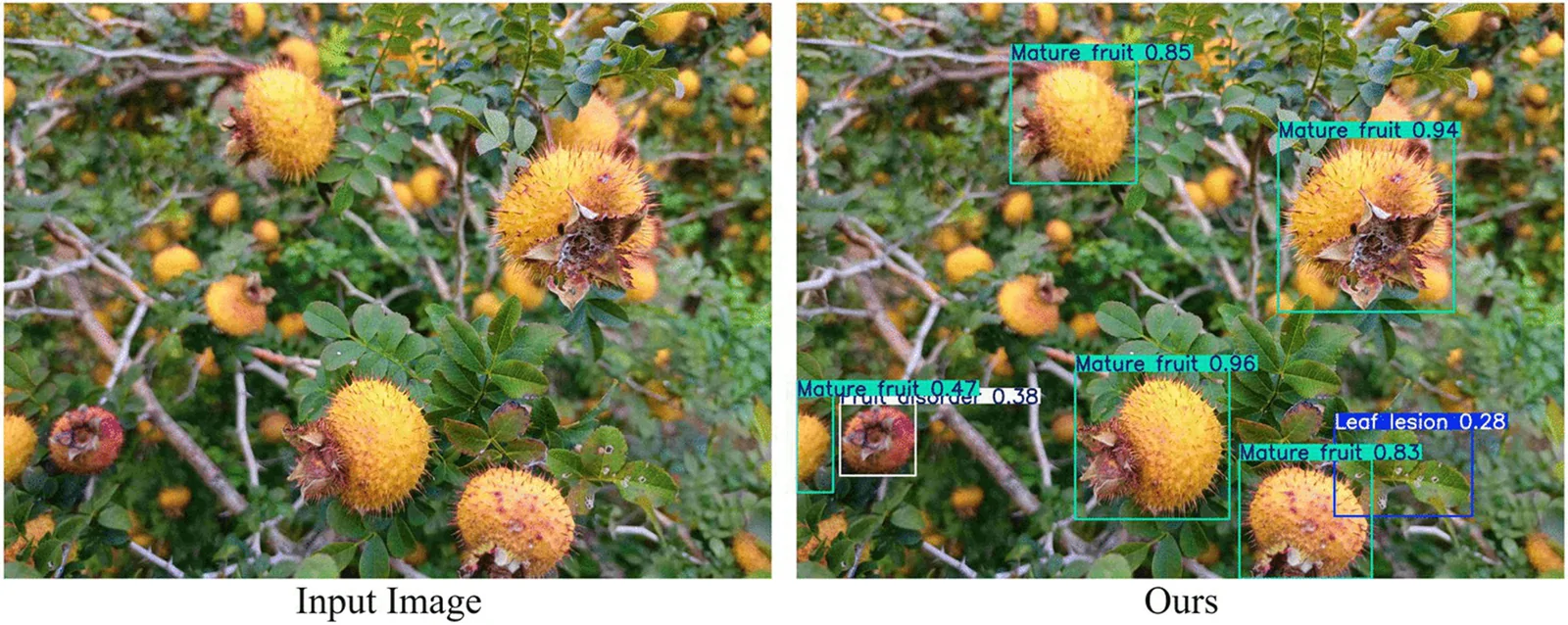
Detection results of *Rosa roxburghii* object dataset using ours models.

**Fig 10:**
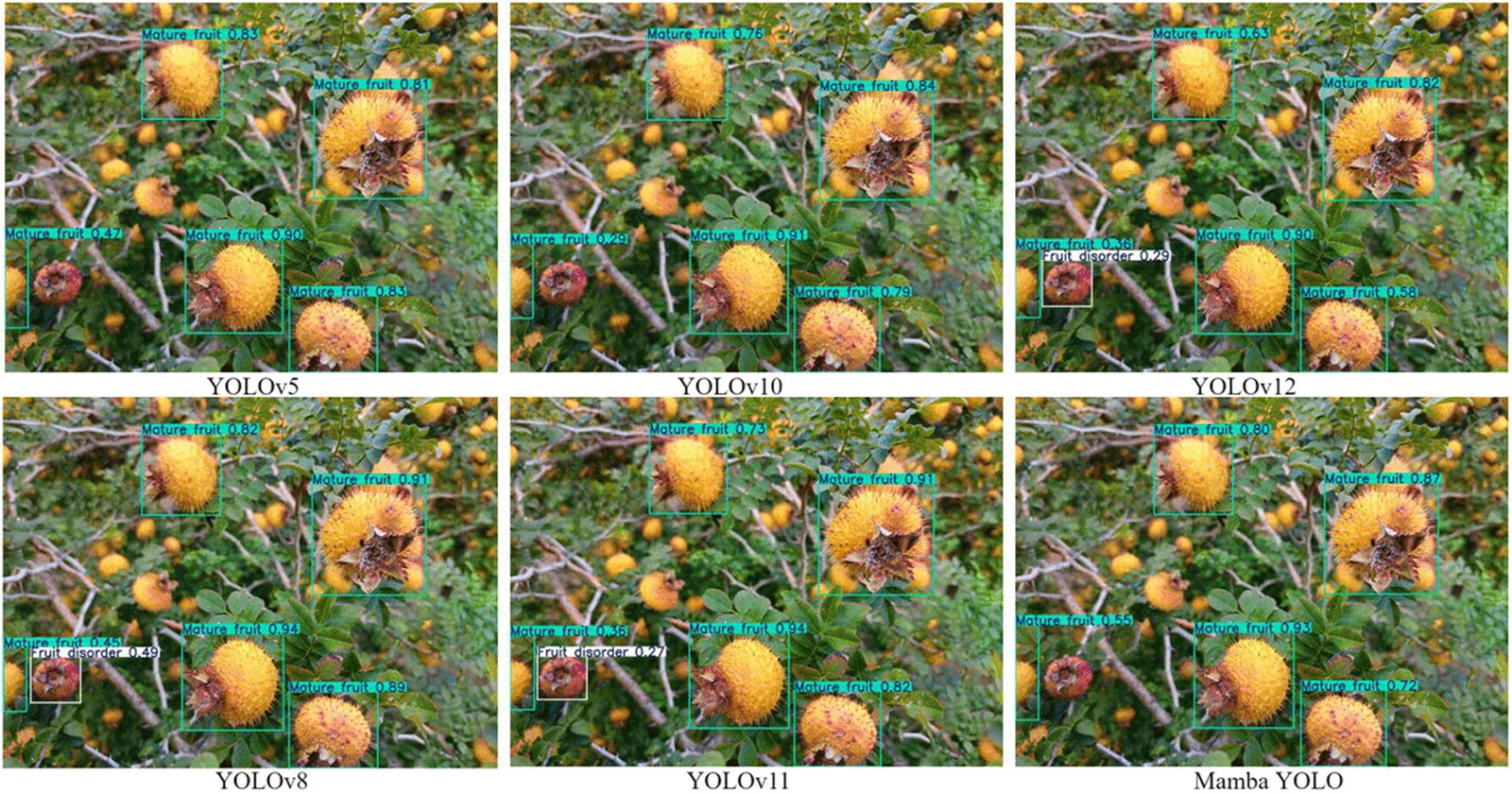
Detection results comparison of *Rosa roxburghii* object dataset using other models.

Fig 11 shows the heat map of *Rosa roxburghii* disease detection using our model, while Fig 12 presents a comparative heat map visualization using other models.To quantitatively support these visual observations, we compute the Attention Concentration Score, defined as the ratio of attention weights within the ground-truth lesion regions to the total attention across the entire image:


$$\begin{array}{c} \text{Score}=\frac{\sum (\text{Attention Map}\times \text{Mask})}{\sum (\text{Attention Map})} \end{array}$$ (14)

where Attention Map denotes the model’s attention weights and Mask denotes the binary ground-truth lesion region. Our YOLO-HMC achieves a score of 0.81, substantially higher than the 0.79 score of YOLOv8, confirming that our model more precisely focuses on disease-relevant regions.The heat map of our model demonstrates that the attention is concentrated primarily on regions relevant to *Rosa roxburghii*, with significantly less focus on irrelevant peripheral areas. In contrast, other models exhibit considerable attention to these marginal irrelevant regions, which leads to a higher number of false detections.Ours model predominantly focuses on the main bodies of *Rosa roxburghii* fruits and salient pathological regions. Through the innovative Synergistic Spatial-Sequential Modeling mechanism, it achieves more precise balancing of feature attention across diverse regions, significantly reducing false edge detections. This architecture enables the model to comprehensively capture multi-scale features and temporal contextual information within images, thereby substantially enhancing detection accuracy.

**Fig 11:**
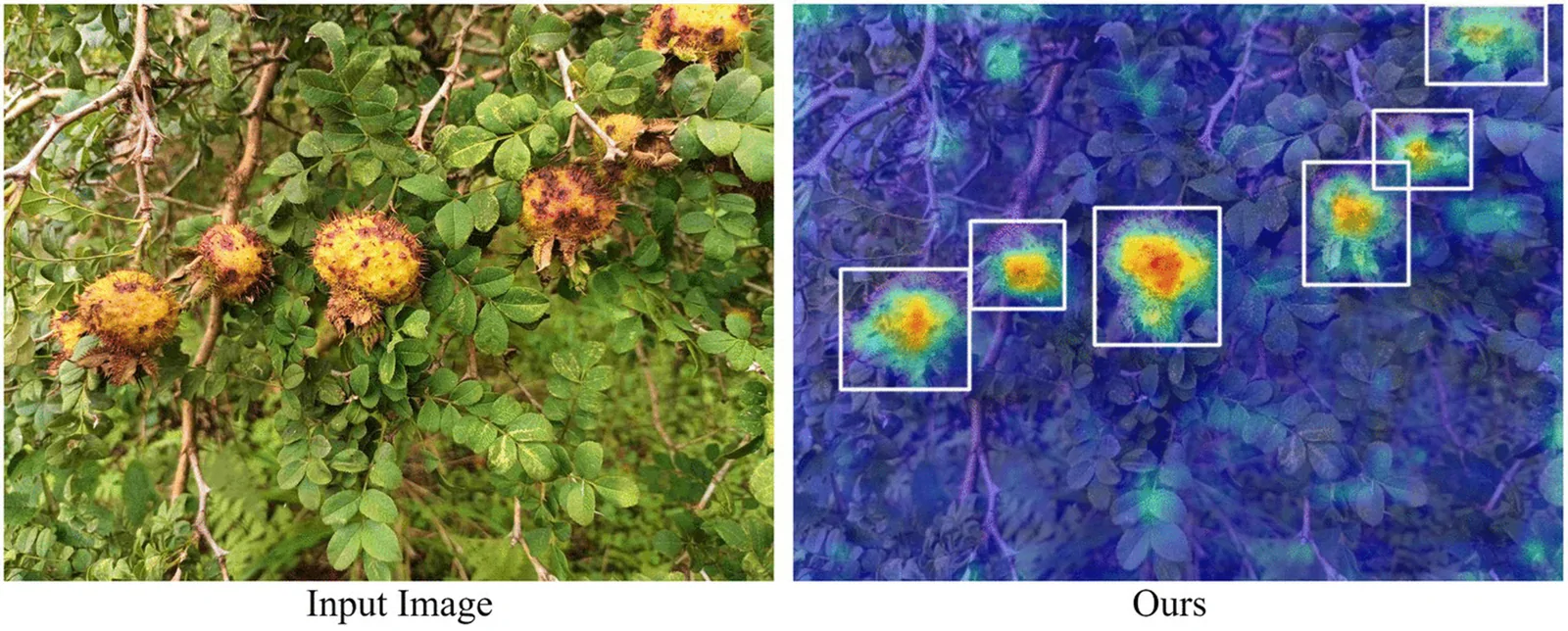
The heat map results of ours model.

**Fig 12:**
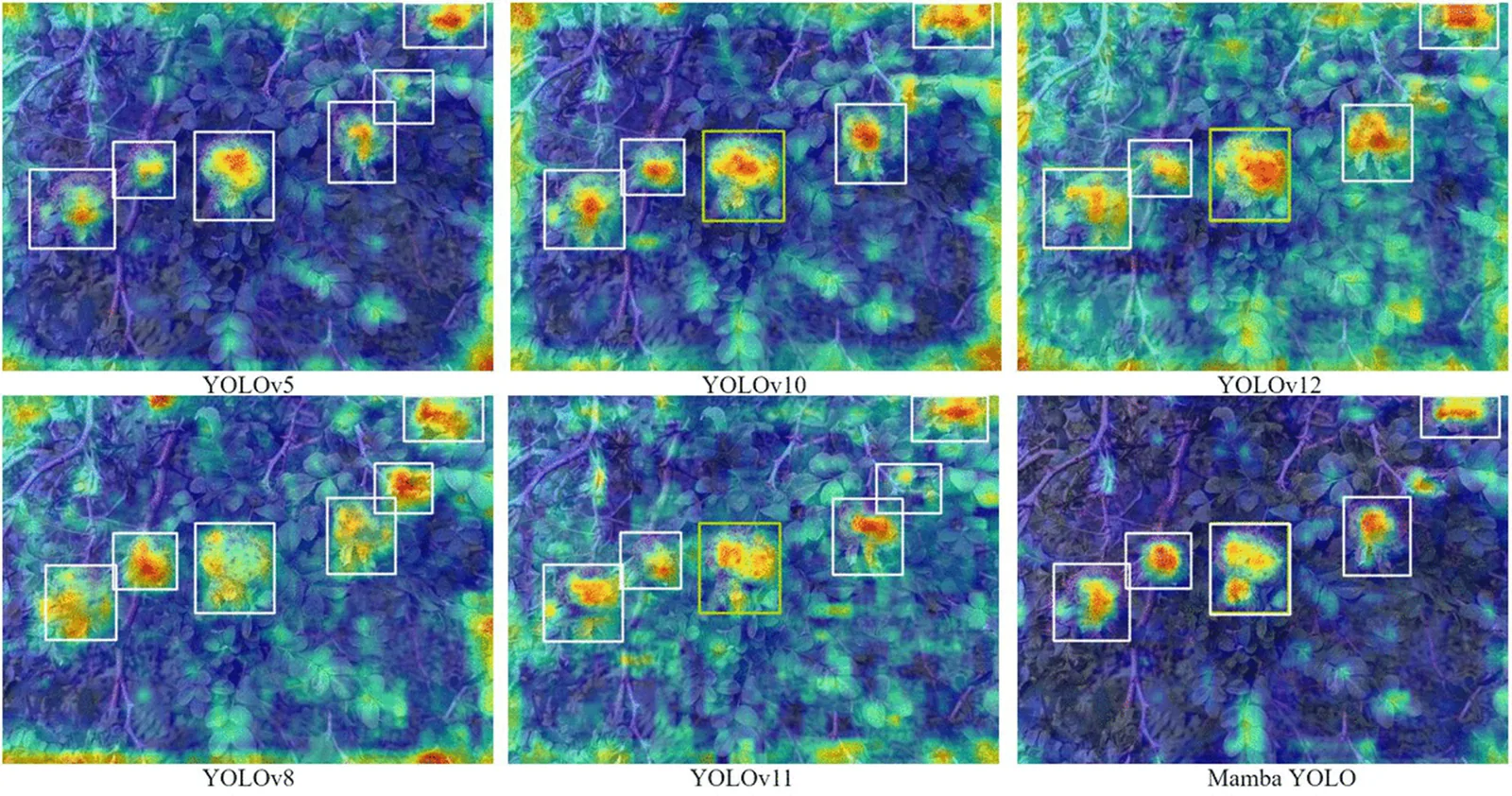
Comparison of heat map from other models.

Table 3 reveals that YOLO-HMC achieves leading performance across different model scales. Compared to the YOLOv8 baseline, the YOLO-HMC N/S/M/L variants registers improvements of 0.27%/5.61%/0.18%/–1.8% in mAP50-95, 2.87%/7.2%/–1.47%/–3.26% in mAP50, and –0.37%/2.51%/0.88%/3.28% in mAP75. The standard deviations across five runs for these improvements are consistently below 0.08% for mAP50-95 and below 0.15% for mAP50 and mAP75, confirming the statistical consistency of our results. These gains come with nearly unchanged parameters and reduced computational load by 2.47%/3.16%/4.45%/5.52%. Compared to other YOLO models, YOLO-HMC-N also achieves superior trade-offs between accuracy and computational cost. YOLO-HMC-N outperforms YOLOv5-N, YOLOv10-N, YOLOv11-N, YOLOv12-N, and Mamba YOLO-T with mAP50-95 gains of 6.85%, 6.99%, 5.19%, 13.66%, and 2.57%, respectively. It also improves mAP50 by 6%, 8.38%, 7.08%, 16.27%, and 6.55%, and mAP75 by 7.55%, 6.7%, 4.36%, 12.45%, and 2.42%. Similarly, YOLO-HMC-S outperforms YOLOv5-S, YOLOv10-S, YOLOv11-S, and YOLOv12-S with mAP50-95 improvements of 6.92%, 17.15%, 5.93%, and 4.36%, mAP50 improvements of 7.78%, 19.38%, 6.29%, and 6.84%, and mAP75 gains of 1.26%, 11.26%, 2.51%, and 0.11%, respectively. Compared to YOLOv5-M, YOLOv10-M, YOLOv11-M, YOLOv12-M, and Mamba YOLO-B, YOLO-HMC-M achieves mAP50-95 gains of 0.93%, 18.46%, 4.82%, 9.8%, and 3.2%, respectively. It also improves mAP50 by 1.32%, 16.99%, 4.41%, 10.2%, and 3.17%, and mAP75 by 0.59%, 16.69%, 4.88%, 8.04%, and 2.42%. Against YOLOv5-L, YOLOv10-L, YOLOv11-L, YOLOv12-L, and Mamba YOLO-L, YOLO-HMC-L showcases mAP50-95 gains of 0.95%, 20.36%, –0.43%, 5.38%, and –0.62%, mAP50 improvements of 1.08%, 17.11%, –1.52%, 3.96%, and –0.8%, and mAP75 gains of 6.25%, 22.9%, 5.57%, 7.89%, and 0.73%, respectively. These comparative results demonstrate that our proposed model consistently outperforms current state-of-the-art methods across all scales.

## Discussion

The seven disease categories in our dataset reflect distinct biological processes: leaf lesions indicate fungal infections, fruit pest infestations reflect direct yield damage, and fruit disorders signal physiological stress. The superior detection performance across these categories suggests strong potential for differentiating biotic and abiotic stresses in real-world cultivation. For practical deployment, our framework can be integrated with mobile platforms to provide real-time diagnostic feedback, enabling targeted interventions and reducing pesticide overuse in mountainous specialty crop production.

## Conclusions

This study introduces YOLO-HMC, an enhanced version of YOLOv8 that combines spatial and sequential modeling to substantially improve accuracy and efficiency in *Rosa roxburghii* image disease detection. The core innovation is the synergistic fusion mechanism where CNN-extracted local spatial features and Mamba-derived global sequential features are bidirectionally enhanced via gated re-weighting, enabling mutual refinement instead of simple aggregation. YOLO-HMC overcomes the shortcomings of conventional CNNs in modeling long-range dependencies and mitigates the computational demands typical of pure Vision Transformers by incorporating advanced features from the Mamba model. The introduction of VSSBlock integrates state space representations with linear-time update mechanisms, enhancing the model’s ability to capture complex disease features in *Rosa roxburghii* images while maintaining computational efficiency on large-scale datasets. Experimental results confirm that YOLO-HMC delivers superior detection performance across seven *Rosa roxburghii* disease categories, including leaf lesions, fruit pest infestations, and fruit disorders—outperforming existing approaches. Ablation studies validate the essential contribution of the VSSBlock in improving model accuracy, expediting convergence, and minimizing computational demands. This work introduces novel technical advancements for intelligent pest and disease monitoring in agriculture and expands the scope of deep learning applications within the sector. For future studies, we will further investigate the capabilities of Mamba models in agricultural image analysis and refine the YOLO-HMC framework to enhance efficiency and precision across diverse crop disease detection tasks. We will also prioritize external validation on independent datasets and cross-domain evaluation to rigorously assess the generalizability and practical applicability of our approach across different agricultural environments and crop types. These efforts will support the intelligent and sustainable advancement of agriculture and related industries.
